# 498 Structural Determination of the CqsR CACHE Domain and its Autoinducer – CORRIGENDUM

**DOI:** 10.1017/cts.2023.551

**Published:** 2023-06-14

**Authors:** Andrew Guarnaccia, Wai-Leung Ng, Anjali Steenhaut, Sandra Olenic, Lark J. Perez, Matthew B. Neiditch

The above abstract [1] published with Wai-Leung Ng’s name spelled incorrectly.

It also published without the middle initials for Lark J. Perez and Matthew B. Neiditch or the proper affiliations.

The correct list of authors and their affiliations is as follows:

Andrew Guarnaccia^1^, Wai-Leung Ng^2^, Anjali Steenhaut^2^, Sandra Olenic^2^, Lark J. Perez^3^, Matthew B. Neiditch^1^

^1^Department of Microbiology, Biochemistry, and Molecular Genetics, New Jersey Medical School, Rutgers, ^2^Department of Molecular Biology and Microbiology, Tufts University School of Medicine, ^3^Department of Chemistry & Biochemistry, Rowan University

The original abstract has been corrected online to rectify these errors.
